# Plasma Levels of Monocyte Chemoattractant Protein-1, n-Terminal Fragment of Brain Natriuretic Peptide and Calcidiol Are Independently Associated with the Complexity of Coronary Artery Disease

**DOI:** 10.1371/journal.pone.0152816

**Published:** 2016-05-12

**Authors:** Roberto Martín-Reyes, Juan Antonio Franco-Peláez, Óscar Lorenzo, María Luisa González-Casaus, Ana María Pello, Álvaro Aceña, Rocío Carda, José Luis Martín-Ventura, Luis Blanco-Colio, María Luisa Martín-Mariscal, Juan Martínez-Milla, Ricardo Villa-Bellosta, Antonio Piñero, Felipe Navarro, Jesús Egido, José Tuñón

**Affiliations:** 1 Department of Cardiology, Instituto de Investigación Sanitaria-Fundación Jiménez Díaz, Madrid, Spain; 2 Laboratory of Vascular Pathology, Instituto de Investigación Sanitaria-Fundación Jiménez Díaz, Madrid, Spain; 3 Autónoma University, Madrid, Spain; 4 Laboratory of Nephrology and Mineral Metabolism, Hospital Gómez-Ulla, Madrid, Spain; 5 Department of Nephrology, Instituto de Investigación Sanitaria-Fundación Jiménez Díaz, Madrid, Spain; 6 CIBERDEM, Madrid, Spain; Children's National Medical Center, Washington, UNITED STATES

## Abstract

**Background and Objectives:**

We investigated the relationship of the Syntax Score (SS) and coronary artery calcification (CAC), with plasma levels of biomarkers related to cardiovascular damage and mineral metabolism, as there is sparse information in this field.

**Methods:**

We studied 270 patients with coronary disease that had an acute coronary syndrome (ACS) six months before. Calcidiol, fibroblast growth factor-23, parathormone, phosphate and monocyte chemoattractant protein-1 [MCP-1], high-sensitivity C-reactive protein, galectin-3, and N-terminal pro-brain natriuretic peptide [NT-proBNP] levels, among other biomarkers, were determined. CAC was assessed by coronary angiogram as low-grade (0–1) and high-grade (2–3) calcification, measured with a semiquantitative scale ranging from 0 (none) to 3 (severe). For the SS study patients were divided in SS<14 and SS≥14. Multivariate linear and logistic regression analyses were performed.

**Results:**

MCP-1 predicted independently the SS (RC = 1.73 [95%CI = 0.08–3.39]; p = 0.040), along with NT-proBNP (RC = 0.17 [95%CI = 0.05–0.28]; p = 0.004), male sex (RC = 4.15 [95%CI = 1.47–6.83]; p = 0.003), age (RC = 0.13 [95%CI = 0.02–0.24]; p = 0.020), hypertension (RC = 3.64, [95%CI = 0.77–6.50]; p = 0.013), hyperlipidemia (RC = 2.78, [95%CI = 0.28–5.29]; p = 0.030), and statins (RC = 6.12 [95%CI = 1.28–10.96]; p = 0.013). Low calcidiol predicted high-grade calcification independently (OR = 0.57 [95% CI = 0.36–0.90]; p = 0.013) along with ST-elevation myocardial infarction (OR = 0.38 [95%CI = 0.19–0.78]; p = 0.006), diabetes (OR = 2.35 [95%CI = 1.11–4.98]; p = 0.028) and age (OR = 1.37 [95%CI = 1.18–1.59]; p<0.001). During follow-up (1.79 [0.94–2.86] years), 27 patients developed ACS, stroke, or transient ischemic attack. A combined score using SS and CAC predicted independently the development of the outcome.

**Conclusions:**

MCP-1 and NT-proBNP are independent predictors of SS, while low calcidiol plasma levels are associated with CAC. More studies are needed to confirm these data.

## Introduction

Increased plasma levels of inflammatory biomarkers have been associated with the development of cardiovascular events. In addition to high-sensitivity C-reactive protein [1], enhanced Monocyte Chemoattractant Protein-1 (MCP-1) plasma levels have been related with adverse outcomes in patients with coronary artery disease (CAD) [2]. Other biomarkers such as galectin-3 and N-terminal fragment of brain natriuretic peptide (NT-proBNP), have demonstrated to be independent predictors of the development of heart failure and death [2–5]

In addition, low vitamin D plasma levels have been independently associated with coronary artery calcification (CAC), atherosclerosis burden, stroke, hypertension, cardiovascular events, and death [6–8]. Furthermore, other components of mineral metabolism are also associated with an enhanced cardiovascular risk [9]. In this regard, increased fibroblast growth-23 (FGF-23), parathormone (PTH) and phosphate plasma levels have been related with cardiovascular damage [10–15].

The SYNTAX score (SS), was developed as a tool to assess the complexity of coronary lesions in the SYNTAX (Synergy between Percutaneous Coronary Intervention with TAXUS and Cardiac Surgery) study [16–18]. Later, this score was seen also to correlate with clinical outcomes [19].

We have investigated the relationship of the SS and the presence of CAC with plasma levels of the components of mineral metabolism and several biomarkers related to cardiovascular damage such as: MCP-1, neutrophil gelatinase-associated lipocalin (NGAL), and soluble tumor necrosis factor-like weak inducer of apoptosis (sTWEAK), involved in inflammation and atherothrombosis [20–24]; galectin-3, related to thrombosis and heart failure [3,25]; and the inactive N-terminal fragment of brain natriuretic peptide (NT-proBNP), related mainly to heart failure [4,5]. High-sensitivity C-reactive protein (hs-CRP) was studied as a reference, given the important amount of information published with this biomarker. In addition, we have assessed the prognostic value of a score combining SS and CAC.

## Methods

### Patients and Study Design

The research protocol complies with the Declaration of Helsinki and was approved by the ethics committees of the participant hospitals. The Ethics Committee for Clinical Research of the Universitary Hospital Fundacion Jimenez Diaz, approved the study. All patients signed informed consent documents. The Biomarkers in Acute Coronary Syndrome (BACS) and Biomarkers in Acute Myocardial Infarction (BAMI) studies, included patients admitted at four hospitals in the area of Madrid with either non-ST elevation acute coronary syndrome (NSTEACS) or ST elevation myocardial infarction (STEMI). Detailed inclusion and exclusion criteria have been reported previously [2]. The present paper analyzes data from patients included in the BACS and BAMI studies at one of the four participant hospitals.

Between July 2006 and April 2010, 676 patients were discharged from Fundación Jiménez Díaz with a diagnosis of NSTEACS or STEMI. Of them, 284 were included in the BACS-BAMI studies. The remaining patients were not included due to: age over 85 years (16.3%), presence of disorders or toxic habits limiting survival (35.8%), impossibility to perform cardiac revascularization (13.5%), coexistence of other significant cardiopathy (5.8%), impossibility to perform follow-up (12.0%), clinical instability beyond the 6th day at the index event (10.1%), refusal to participate in the study (0.9%), death before the second visit (0.3%), and impossibility of the investigators to include them (5.3%). Finally, 14 patients had not coronary angiogram available, leading 270 patients for analysis.

Patients were seen six months later, on an outpatient basis. Plasma was withdrawn and a complete set of clinical variables was recorded. This study reports clinical and analytical findings obtained at this outpatient visit relating them to subsequent follow-up.

### Laboratory Determinations

The investigators who performed the analytical studies were unaware of clinical data.

Plasma concentrations of MCP-1, galectin-3, sTWEAK, and NGAL were determined in duplicate using commercially available enzyme-linked immunosorbent assay kits (BMS279/2, BenderMedSystems, Burlingame, California; DCP00, R&D Systems, Minneapolis, Minnesota; BMS2006INST, Bender MedSystems, Burlingame, California; and Kit 036, BioPorto, Gentofte, Denmark, respectively) following the manufacturers’ instructions. Intra- and interassay coefficients of variation were 4.6% and 5.9% for MCP-1, 6.2% and 8.3% for galectin-3, 6.1% and 8.1% for sTWEAK, and 5.3% and 7.9% for NGAL, respectively. High-sensitivity C-reactive protein was assessed by latex-enhanced immunoturbidimetry (ADVIA 2400 Chemistry System; Siemens, Munich, Germany) and NT-proBNP by immunoassay (VITROS; Ortho Clinical Diagnostics Raritan, New Jersey). Lipid, glucose, and creatinine levels were determined by standard methods (ADVIA 2400 Chemistry System; Siemens).

Plasma levels of calcidiol (a vitamin D metabolite) were quantified by chemiluminescent immunoassay (CLIA) on the LIAISON XL analyzer (LIAISON 25OH-Vitamin D total Assay DiaSorin, Saluggia, Italy), FGF-23 was measured by an enzyme-linked immunosorbent assay which recognizes epitopes within the carboxyl-terminal portion of FGF- 23 (Human FGF-23, C-Term, Immutopics Inc, San Clemente, CA), intact PTH was analyzed by a second-generation automated chemiluminescent method (Elecsys 2010 platform, Roche Diagnostics, Mannheim, Germany) and phosphate was determined by an enzymatic method (Integra 400 analyzer, Roche Diagnostics, Mannheim, Germany).

### Assessment of Syntax Score and Coronary Calcium

SS and CAC were assessed by two interventional cardiologists. SS was calculated using the SS web calculator version 2.11 and CAC was classified from 0 to 3 using a scale where 0 was absence of CAC, 1 mild (radiopacities noted only during the cardiac cycle before contrast injection after carefully examination), 2 moderate (evident radiopacities noted only during the cardiac cycle before contrast injection), and severe (radiopacities noted without cardiac motion before contrast injection generally compromising both sides of the arterial lumen), [26].

To simplify the analysis, a binary calcification (BC) variable (low [0–1] and high [2–3] grade calcification) was created and Kappa coefficient was performed to assess the inter-observer agreement. Finally, to assess the prognostic value of CAC and SS a score combining these two variables was designed. Patients were divided in three groups: Group 0 (SS below the median of global cohort and BC 0–1), Group 1 (SS above or equal the median or BC 2–3) and Group 2 (SS above or equal the median and BC 2–3).

### Definition of Events

Patients were considered to present hypertension if they had a history of systolic and/or diastolic pressure equal to or higher than 140 and 90 mm Hg, respectively or if they were taking blood-pressure-lowering drugs for this disorder. Patients with current or past tobacco use were considered smokers. Patients receiving lipid-lowering therapy for this diagnosis and those with fasting lipid levels [LDL cholesterol>160 mg/dl and/or triglyceride levels>200 mg/dl] were considered to be diagnosed with dyslipidemia. Finally, patients were considered to be diabetics if they were receiving therapy for the disease or if they had fasting glucose levels > 126 mg/dl.

After the outpatient visit, patients were followed recording the incidence of the outcome composed of NSTEACS, STEMI, and stroke/transient ischemic attack, defined as described previously [2].

### Statistical methods

Quantitative data following a normal distribution are presented as mean +/- SD and compared using the Student t-test or ANalysis Of VAriance (ANOVA). Those not normally distributed are displayed as median (interquartile range) and compared using the Mann-Whitney test. Qualitative variables are displayed as percentages and compared using chi-square or Fisher’s exact test when appropriate.

All variables were analyzed by linear regression taking the presence of SS as dependent variable, and by binary logistic regression taking CAC as dependent variable. Thereafter, we constructed a multivariate model including all variables that had a level of significance p<0.2 at univariate analyses. The final model included all variables with p<0.05.

Survival-free of outcome curves were traced with the Kaplan-Maier method and groups were compared with the log-rank test. Cox proportional hazards model was used with forward stepwise selection to assess the variables associated with the outcome. All the variables with p<0.2 were included into the multivariate model. This multivariate analysis was done with the backward step method.

Analyses were performed with SPSS 19.0 (SPSS Inc., New York), and were considered significant when p<0.05 (two-tailed).

## Results

Baseline characteristics, clinical variables and plasma biomarkers levels are listed in Table 1. Stratification by age showed that MCP-1, Galectin-3, NT-proBN,P NGAL, and PTH plasma levels increased along with age (S1 Table). Women were older than men [(71.5 (59.0–80.0) vs 62.0 (52.2–74.7) years; p<0.001)] and had higher plasma levels of Galectin-3, PTH, and phosphate (S1 Table).

**Table 1:** Baseline characteristics, clinical variables and plasma biomarkers levels.

Variable	Total population (N = 270)	SS (<14)131 patients	SS (≥14)139 patients	p value	BC (0–1)219 patients	BC (2–3)51 patients	p value
Age (yrs)	65.0 (54.0–76.0)	63.0 (51.0–73.0)	71.0 (58.0–78.0)	**<0.001**	63.0 (52.0–75.0)	76 (62.0–81.0)	**<0.001**
Men	180 (66.7%)	79 (60.3%)	101 (72.7%)	**0.031**	150 (68.5%)	30 (58.8%)	0.193
Body mass index (kg/m^2^)	27.9 (25.5–30.9)	28.2 (25.7–31.6)	27.7 (25.0–30.1)	0.345	28.1 (25.6–31.0)	27.3 (24.2–30.0)	0.283
Diabetes mellitus	56 (20.7%)	22 (16.8%)	34 (24.5%)	0.119	40 (18.3%)	16 (31.4%)	**0.045**
Smoker (present or former)	186 (68.9%)	91 (69.5%)	95 (68.3%)	0.842	155 (70.8%)	31 (60.8%)	0.172
Hypertension	189 (70%)	80 (61.1%)	109 (78.4%)	**0.002**	148 (67.6%)	41 (80.4%)	0.063
Dyslipidemia	135 (50%)	61 (46.6%)	74 (53.2%)	0.273	111 (50.7%)	24 (47.1%)	0.641
Peripheral artery disease	4 (1.5%)	0 (0%)	4 (2.9%)	0.123	1 (0.5%)	3 (5.9%)	**0.022**
Cerebrovascular events	15 (5.6%)	4 (3.1%)	11 (7.9%)	0.075	10 (4.6%)	5 (9.8%)	0.170
Atrial fibrillation	17 (6.3%)	8 (6.1%)	9 (6.5%)	0.901	13 (5.9%)	4 (7.8%)	0.537
LV ejection fraction <40%	60.0% (50.0–67.0)	60.0% (52.0–69.0)	59.0% (45.0–65.5)	**0.013**	60.0 (50.0–68.0)	60.0 (45.0–66.0)	0.557
ASA	246 (91.1%)	123 (93.9%)	123 (88.5%)	0.115	202 (92.2%)	44 (86.3%)	0.179
Clopidogrel	202 (74.8%)	95 (72.5%)	107 (77.0%)	0.399	163 (74.4%)	39 (76.5%)	0.761
Acenocumarol	16 (5.9%)	6 (4.6%)	10 (7.2%)	0.360	13 (5.9%)	3 (5.9%)	1.000
Statins	251 (93%)	120 (91.6%)	131 (94.2%)	0.396	202 (92.2%)	49 (96.1%)	0.543
ACEI	187 (69.3%)	93 (71.0%)	94 (67.6%)	0.549	157 (71.7%)	30 (58.8%)	0.079
ARB	57 (21.1%)	23 (17.6%)	34 (24.5%)	0.164	46 (21.0%)	11 (21.6%)	0.929
β-Blockers	191 (70.7%)	90 (68.7%)	101 (72.7%)	0.475	154 (70.3%)	37 (72.5%)	0.751
STEMI*	126 (46.7%)	59 (45.0%)	67 (48.2%)	0.603	111 (50.7%)	15 (29.4%)	**0.005**
SYNTAX score*	14.0 (7.0–23.0)	6.5 (3.0–10.0)	23.0 (17.5–28.0)	**<0.001**	12.0 (6.0–21.5)	22.0 (16.0–33.0)	**<0.001**
BC (0–1) %/ BC (2–3) %	81.1/18.9	93.1/6.9	69.8/30.2	**<0.001**	------------------------------	-----------------------------	**-------**
Complete revascularization*	189 (70%)	107 (81.7%)	82 (59.0%)	**<0.001**	160 (73.1%)	29 (56.9%)	**0.027**
LDL cholesterol (mg/dl)	75.5 (61.0–94.0)	77.0 (64.0–95.0)	74.0 (59.0–93.0)	0.338	76.0 (61.0–93.0)	74.0 (61.0–101.0)	0.862
HDL cholesterol (mg/dl)	44.0 (36.0–51.0)	45.0 (36.0–56.0)	43.0 (37.0–47.0)	0.077	44.0 (36.0–51.0)	44.0 (38.0–50.0)	0.575
Triglycerides (mg/dl)	102.0 (74.7–139.2)	97.0 (75.0–132.0)	107.0 (74.0–150.0)	0.238	101.0 (76.0–142.0)	109.0 (65.0–138.0)	0.666
eGFR (ml/min/1.73 m^2^)	75.2 (60.1–87.4)	80.3 (65.3–91.7)	70.0 (56.0–83.1)	**<0.001**	76.7 (62.3–89.9)	66.4 (51.1–82.0)	**0.010**
hsCRP (mg/L)	1.7 (0.7–3.7)	1.6 (0.6–3.4)	1.8 (0.8–4.1)	0.271	1.8 (0.7–3.8)	1.4 (0.7–3.6)	0.601
MCP-1 (pg/ml)	142.4 (112.2–179.2)	130.5 (109.0–165.6)	153.7 (119.8–188.1)	**0.001**	137.0 (110.1–176.5)	165.6 (128.1–197.0)	**0.005**
Galectin-3 (ng/ml)	8.6 (7.2–10.0)	8.3 (7.2–9.7)	8.7 (7.2–10.6)	0.058	8.4 (7.0–9.8)	9.0 (7.5–10.5)	0.078
NT-proBNP (pg/ml)	209.5 (99.5–521.0)	151.0 (85.4–289.0)	343.0 (125.0–848.0)	**<0.001**	167.0 (88.7–450.0)	387 (176.0–860.0)	**0.001**
NGAL (ng/ml)	165.2 (127.3–219.2)	153.9 (119.5–202.6)	178.4 (131.0–238.8)	**0.002**	156.5 (121.7–213.8)	195.9 (161.5–247.6)	**0.002**
sTWEAK (pg/ml)	198.0 (159.4–249.2)	200.3 (160.9–267.2)	196.7 (156.2–244.7)	0.245	201.5 (157.3–264.8)	193.7 (167.8–228.8)	0.283
PTH (pg/ml)	65.1 (49.5–84.2)	60.8 (47.4–81.1)	68.7 (51.1–90.9)	**0.039**	63.3 (48.9–82.5)	71.7 (51.3–96.0)	**0.037**
Phosphate (mg/dl)	3.3 (2.9–3.6)	3.3 (3.0–3.7)	3.2 (2.8–3.6)	0.133	3.2 (2.9–3.6)	3.3 (3.0–3.7)	0.317
AP (UI/l)	75 (61.5–92.5)	76 (62.7–93)	74 (61–92)	0.747	74 (61–92)	79 (64–96)	0.544
FGF-23 (RU/ml)	68.1 (54.4–88.8)	67.0 (54.7–86.8)	69.3 (54.4–92.9)	0.650	67.0 (52.3–87.2)	72.0 (13.0–25.8)	0.124
Calcidiol (ng/ml)	18.4 (12.5–24.9)	18.8 (13.0–25.8)	16.5 (11.7–24.0)	0.134	18.8 (13.0–25.8)	14.6 (10.2–20.9)	**0.007**

Quantitative data following a normal distribution are presented as mean (standard deviation [SD]) and those not normally distributed are displayed as median (interquartile range). LV: left ventricle, ASA: Acetylsalicylic acid, ACEI: Angiotensin-converting enzyme inhibitors, ARB: Angiotensin-receptor blockers, STEMI: ST-elevation myocardial infarction, BC: Binary Calcification, LDL: Low-density lipoprotein, HDL: High-density lipoprotein. eGFR: Glomerular Filtration Rate as estimated by CKD-EPI (Chronic Kidney Disease Epidemiology Collaboration), Hs-CRP: High-sensitivity C-reactive protein, MCP-1: Monocyte chemoattractant protein-1. NGAL: Neutrophil gelatinase-associated lipocalin. sTWEAK: Soluble tumor necrosis factor-like weak inducer of apoptosis, PTH: Parathormone, AP: Alkaline Phosphatase. FGF-23: Fibroblast growth factor-23.

* Data related to the previous acute coronary syndrome

### Biomarkers associated with a high Syntax Score

Median SS was 14 (7–23). There were 198 cases with a SS 0–22, 50 with 23–32 and 22 with≥33. For descriptive purposes we divided the total population into two similar groups according to the median: SS<14 (131 patients) and SS≥14 (139 patients). S2 Table shows the results of the univariate analysis.

At multivariate regression analysis (Table 2), MCP-1 was an independent predictor of SS (RC [Regression Coefficient] = 1.73 for each increase of 50 pg/mL, [95%CI = 0.08–3.39]; p = 0.040) along with NT-proBNP (RC = 0.17 for each increase of 100 pg/mL, [95% CI = 0.05–0.28]; p = 0.004), male sex (RC = 4.15 [95%CI = 1.47–6.83]; p = 0.003), age (RC = 0.13 for each increase of 5 years, [95%CI = 0.02–0.24]; p = 0.020), hypertension (RC = 3.64 [95%CI = 0.77–6.50]; p = 0.013), hyperlipidemia (RC = 2.78 [95%CI = 0.28–5.29]; p = 0.030) and statins (RC = 6.12 [95%CI = 1.28–10.96]; p = 0.013).

**Table 2:** Multivariate regression analysis showing the variables independently associated with SS and CAC.

**SS as dependent variable**	**Regression Coefficient**	**95% CI Lower**	**Upper**	**P**
Age (yrs)*	0.13	0.02	0.24	0.020
Men	4.15	1.47	6.83	0.003
Hypertension	3.64	0.77	6.50	0.013
Hyperlipidemia	2.78	0.28	5.29	0.030
MCP-1**	1.73	0.08	3.39	0.040
NT-ProBNP***	0.17	0.05	0.28	0.004
Statins	6.12	1.28	10.96	0.013
**CAC as dependent variable**	**Odds Ratio**	**95% CI Lower**	**Upper**	**P**
Age (yrs)*	1.37	1.18	1.59	<0.001
Diabetes Mellitus	2.35	1.11	4.98	0.028
STEMI	0.38	0.19	0.78	0.006
Calcidiol****	0.57	0.36	0.90	0.013

**Abbreviations:** SS: Syntax Score, CAC: Coronary artery calcification, CI: Confidence interval, other abbreviations as for Table 1.

* Age: RC or OR for each increase of 5 years.

** MCP-1: RC for each increase of 50 pg/mL.

*** NT-proBNP: RC for each increase of 100 pg/mL.

****Calcidiol: OR for each increase of 10 ng/mL.

### Predictors of coronary calcification

Fifty-one patients (18.9%) had high grade BC (2–3). The kappa coefficient for inter-observer agreement was 0.783. S3 Table displays the results of the univariate logistic regression analysis.

At multivariate regression analysis (Table 2), calcidiol was the only biomarker that was shown to be an independent predictor of BC (OR [Odds Ratio] = 0.57 for each increase of 10 ng/mL, [95%CI = 0.36–0.90]; p = 0.013). Other independent predictors were STEMI (OR = 0.38,[95%CI = 0.19–0.78]; p = 0.006), diabetes (OR = 2.35, [95%CI = 1.11–4.98]; p = 0.028) and age (OR = 1.37 for each increase of 5 years, [95%CI = 1.18–1.59]; p<0.001).

### Prognostic Value of a combined SS and calcium score

Patients were divided in three groups according to a combined score including SS and BC, as explained previously. The distribution of clinical variables across the different subgroups is shown in Table 3.

**Table 3:** Distribution of clinical variables across the different subgroups according to the combined Syntax Score and Calcium Score.

Variable	Total population (N = 270)	Group 0 (SS<14 and BC 0–1)(N = 122)	Group 1 (SS ≥14 or BC 2–3)(N = 106)	Group 2 (SS ≥14 and BC 2–3)(N = 42)	P value
Age (yrs)	65.0 (54.0–76.0)	62 (51–73)	66.5 (55–77)	76.5 (65–81)	**0.001**
Men	66.7%	60.7%	76.4%	59.5%	0.441
Body mass index (kg/m^2^)	27.9 (25.5–30.9)	28.5 (25.8–31.7)	27.7 (25.1–30.2)	27.7 (24.6–30.1)	0.352
Diabetes mellitus	20.7%	15.6%	22.6%	31%	**0.028**
Smoker (present or former)	68.9%	70.5%	69.8%	61.9%	0.375
Hypertension	70%	59%	79.1%	78.6%	**0.002**
LV ejection fraction <40%	60.0% (50.0–67.0)	60.0% (52.0–70.0)	57.0% (46.0–64)	60% (45–67)	**0.017**
ASA	91.1%	95.9%	85.8%	90.5%	**0.025**
Clopidogrel	74.8%	71.3%	79.2%	73.8%	0.383
Acenocumarol	5.9%	7.9%	6.6%	7.1%	0.811
Statins	93%	91.8%	92.5%	97.6%	0.351
β-Blockers	70.7%	68.9%	71.7%	73.8%	0.802
STEMI	46.7%	45.1%	56.6%	26.2%	**0.030**
Complete revascularization	70%	81.1%	65.1%	50%	**0.001**
eGFR (ml/min/1.73 m^2^)	75.2 (60.1–87.4)	80.5 (66.6–91.8)	71.9 (57.4–85.2)	66.3 (52.2–80.5)	**0.001**

Abbreviations, as for Table 1.

**Fig 1:**
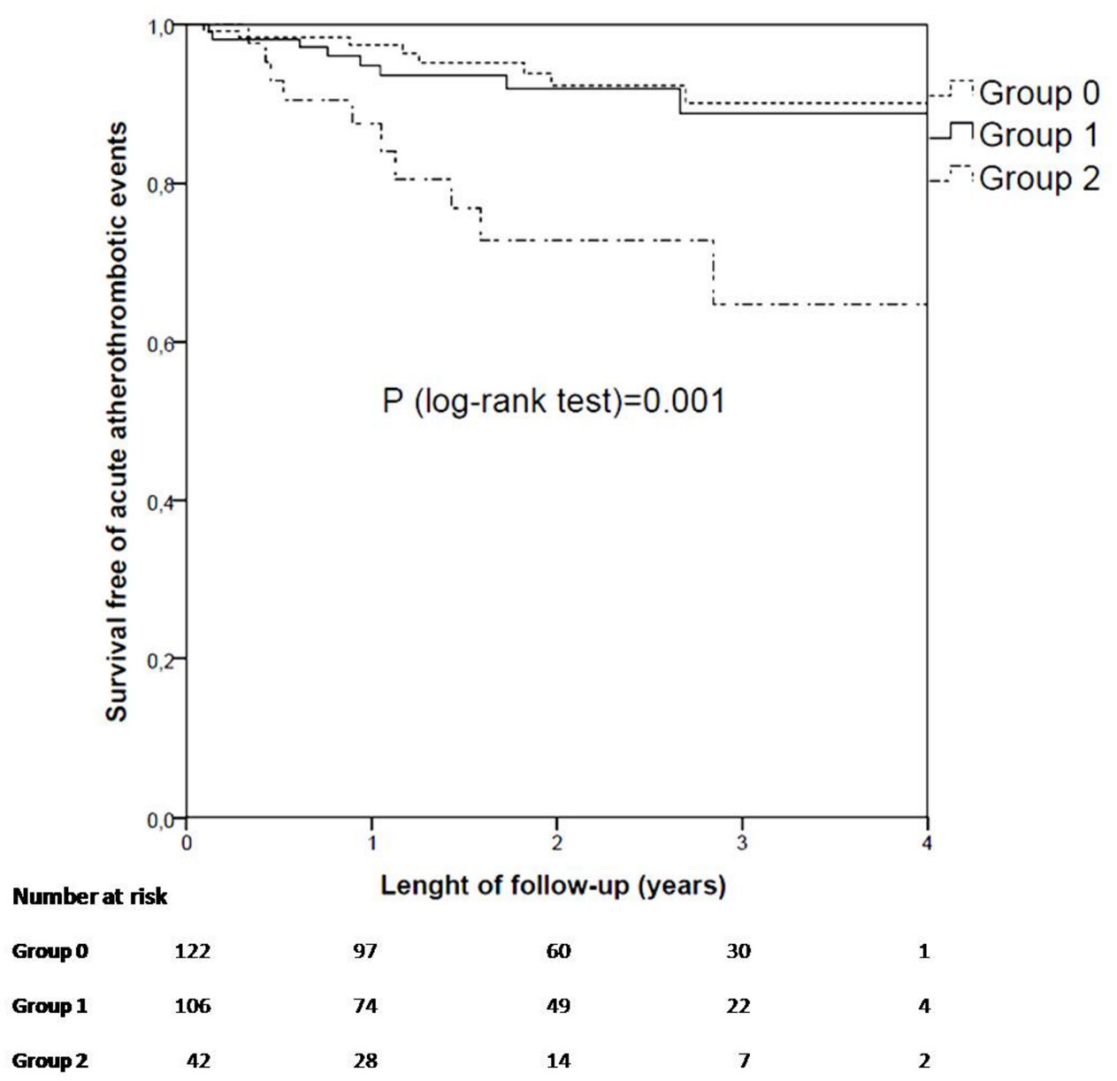
Kaplan-Meier curves showing time to outcome according to the combined syntax and calcium score.

During a median follow-up of 1.79 (0.94–2.86) years, a total of 27 patients reached the outcome. Group 0 (122 patients): 8 events (6.6%), Group 1 (106 patients): 9 events (8.5%), and Group 2 (42 patients): 10 events: (23.8%). Kaplan-Maier curves show that patients from group 2 had a lower event-free survival (p = 0.001) (Fig 1) than the other two groups.

S4 Table displays the results of the univariate Cox regression analysis.

At multivariate analysis, we found that the only independent predictors were the combined variable of SS and CC (HR [Hazard Ratio] (group 2 vs. group 0) = 4.83 [95%CI = 1.90–12.27]; p = 0.001; HR (group 1 vs. group 0) = 1.35 [95%CI = 0.51–3.62]; p = 0.549; HR (group 2 vs. group 1) = 3.57 [95%CI = 1.40–9.08]; p = 0.008) and body-mass index (HR = 1.08 [95% CI = 1.01–1.15] for each increase of 1 kg/m^2^, p = 0.041). None of the biomarkers studied in our work were found to be independent predictors of outcomes.

## Discussion

### MCP-1 and NT-proBNP as predictors of the complexity of CAD

The SS is a scoring system that ranks the complexity of the coronary anatomy and is useful to make decisions regarding coronary revascularization [16,18,27], having also prognostic ability [18,19,28]. This makes sense, as the SS takes into account the number, extension, and severity of lesions, and the existence of thrombus or collateral vessels and if the lesion is located in tortuous vessel areas [27]. Then, it adds a lot of characteristics to the simple definition of the number of diseased vessels that are related with the severity of CAD, and with the probability of receiving an adequate revascularization.

In our paper we demonstrate, for the first time, that high MCP-1 levels predict independently the existence of a high SS. This is a chemokine that plays a key role in the recruitment of monocytes into the vascular wall during atherogenesis [20,21]. In fact, we have demonstrated that drugs that interfere with the progression of atherosclerosis, such as statins and angiotensin-converting enzyme inhibitors, decrease the expression of this chemokine [29,30]. Even more, MCP-1 may have procoagulant properties [31]. In accordance with this, MCP-1 plasma levels predict the risk of cardiovascular events in patients with stable CAD [2] and acute coronary syndrome [32,33]. Therefore, the ability of MCP-1 to predict the complexity of CAD fits with its known properties.

On the other hand, increased NT-proBNP plasma levels were also independent predictors of a high SS, even after controlling for the existence of important left ventricular dysfunction in the multivariate analysis. In this case, the potential mechanism of this association is not clear. However, NT-proBNP may have unexpected roles. In this regard, although initially described in the brain, it is now known to be secreted by the ventricular myocardium [5], and it is being used for the diagnosis of heart failure [4,5], having also prognostic value in patients with cardiovascular disease [2]. More recently, NT-proBNP levels have been shown to increase in patients with cancer [34–37]. Given that natriuretic peptides have been shown to have antiproliferative effects [38–40] it has been hypothesized that NT-proBNP plasma levels could increase in response to the excessive cell proliferation present in cancer [37]. Then, the possibility exists that NT-proBNP plasma levels could also increase in response to the cell proliferation present in atherosclerosis, and that theoretically should be especially important in cases of extensive and severe CAD. Nevertheless, more research is needed to elucidate the link between natriuretic peptides and the existence of a high SS.

Finally, there was a positive association between statin therapy and SS. However, this was an observational study and the therapy with statins recorded reflects the treatment given to the patients after the coronary angiogram, not in the previous years where the complexity of CAD was established. Thus, we estimated that this fact simply suggests that patients with more severe CAD were receiving statins more often.

### Low levels of calcidiol predict the existence of high-grade calcification in CAD

CAC is known as sub-clinical atherosclerosis, and has prognostic implications [41]. Moreover, severe CAC limits stent expansion, a fact that can be associated with restenosis and stent thrombosis [42].

In the last years there has been growing interest in the relationship of mineral metabolism with cardiovascular disease. Classically, mineral metabolism has been related to chronic kidney disease. In this regard, when kidneys begin to fail, phosphate excretion decreases, and increased phosphatemia may be associated to cardiovascular damage [13]. To compensate this, there is an increase in plasma levels of FGF-23 and PTH, which promote phosphaturia [10]. However, enhanced FGF-23 and PTH levels may also favor vascular damage, and left ventricular hypertrophy [10–15]. In addition, FGF-23 decreases vitamin D availability by both increasing its catabolism and diminishing its synthesis [10]. Low levels of vitamin D may lead to endothelial dysfunction, inflammation, activation of the renin-angiotensin system, vascular smooth muscle cell proliferation and CAC [6–14,43–45]. In accordance with this, low vitamin D levels have been associated with a high risk of cardiovascular disease and cardiovascular death in patients with and without cardiovascular disease at baseline [15,46–48].

Although low vitamin D levels are characteristic of chronic kidney disease, we have described recently that more than 50% of patients with CAD present low levels of calcidiol, a metabolite of vitamin D, in the presence of average estimated glomerular filtration rate [15]. Even more, low calcidiol levels and other abnormalities of mineral metabolism were evident in the presence of mild decreases in estimated glomerular filtration rate.

In our study, low calcidiol levels were independently associated with high grade CAC. This could play an important role in the higher incidence of adverse outcomes seen in patients with low calcidiol levels. Accordingly, Watson et al found an inverse relationship between 1,25-vitamin D plasma levels and CAC [7]. However, they studied patients at high risk of CAD in primary prevention. We have confirmed these findings in patients with CAD. Moreover, we performed a complete study of mineral metabolism including FGF-23, PTH, alkaline phosphatase, and phosphate plasma levels. Interestingly, the inverse association between vitamin D and CAC was evident despite controlling for levels of all these molecules. This suggests that vitamin D may have a beneficial effect on the process of coronary calcification that may be independent of other abnormalities of mineral metabolism.

### Prognostic value of a score combining CAC and SS

The SS and CAC are two variables related with the extent and severity of the CAD. Even more, they have been shown to predict clinical events [19,28,41]. Our study suggests that a scale combining SS and BC outperforms these isolated scores. Of interest, this finding was obtained after controlling for a large set of clinical variables and more important, biomarkers that have been shown previously to have a good prognostic value, such as MCP-1 and NT-proBNP [2,32,33]. Thus, this finding should be tested in future studies involving larger populations as these biomarkers could be acting as surrogates of the coronary anatomy complexity. So, it could be more effective to use this score than performing biomarker determinations.

### Limitations

Plasma determination was performed six months after coronary angiogram was carried out. Nevertheless, the development of CAC and of complex coronary lesions is a chronic process. Analyzing data in the setting of the previous acute coronary syndromes, could have given confounding results, as biomarker levels may change in the acute episode. Thus, studying biomarkers with the patients in a stable situation may give a more accurate idea of their chronic levels. The therapy used in these patients could theoretically influence levels of the biomarkers studied. However, we have taken this into account in our analyses, and there was no influence of the treatment used.

Finally, given the limited sample size and number of events, these results should be confirmed in future studies.

## Conclusions

Increased MCP-1 and NT-proBNP plasma levels are independently associated with high SS. In addition, low calcidiol levels are related to high grade CAC, even when other components of mineral metabolism are taken into account. Although the relationship between inflammation and atherosclerosis is clear, more work is needed to ascertain the cause of the possible relationship of NT-proBNP with a high SS. Finally, a score combining CAC and SS, but not the biomarkers studied, has independent prognostic value. Thus, coronary anatomy should be taken in consideration in future biomarkers studies to rule out the possibility that the predictive value of these molecules could be a surrogate of the existence of complex coronary lesions.

## Appendix

The following persons participated in blood extraction, plasma isolation, biobank organization or occasional patient recruitment: Pedro Almeida, MD, PhD, Javier Higueras, MD PhD, Rosario De Nicolás, LT, Dolores Asensio, MD, Pilar Jiménez-Caballero, RN, Marta Hernán-Bru, RN, Esmeralda Serrano-Blázquez, RN, Ana Encinas-Pastor, RN, Arantxa Garciandia, RN, Consuelo Ceballos, RN, Belén Arribas, RN and José De la Paz, LT.

## Supporting Information

S1 Table Levels of the different biomarkers distributed by age and sex. (DOCX)

S2 Table Univariate linear regression analysis for prediction of Syntax Score. (DOCX)

S3 Table Univariate logistic regression analysis for prediction of Coronary Artery Calcification. (DOCX)

S4 Table Univariate Cox regression analysis for prediction of AAE. (DOC)
