# Supplementary material for: Plasma Levels of Monocyte Chemoattractant Protein-1, n-Terminal Fragment of Brain Natriuretic Peptide and Calcidiol Are Independently Associated with the Complexity of Coronary Artery Disease
Source: PLoS One. 2016 May 12;11(5):e0152816. doi: 10.1371/journal.pone.0152816 (PMC4865225; doi:10.1371/journal.pone.0152816)
Supplement: S1 Table — (DOCX) [file pone.0152816.s001.docx]

| **Variable** | **<55 years** | **55-70 years** | **>70 years** | **p** |  | **Female** | **Male** | **p** |
| --- | --- | --- | --- | --- | --- | --- | --- | --- |
| **MCP1** | 117.5 (98.2-150.6) | 137.6 (110.6-179.1) | 164.6 (134.7-197.8) | <0.001 |  | 142.4 (116.2-186.0) | 142.6 (110.1-179.2) | 0.645 |
| **Gal3** | 7.4 (6.1-8.8) | 8.5 (7.4-9.3) | 9.5 (8.2-10.9) | <0.001 |  | 9.1 (7.9-10.7) | 8.3 (6.7-9.7) | <0.001 |
| **ProBNP** | 92.3 (54.5-159.0) | 160.5 (90.1-287.7) | 454.0 (222.7-1232.5) | <0.001 |  | 271.0 (141.5-674.5) | 164.5 (87.1-462.2) | 0.433 |
| **NGAL** | 150.8 (117.4-207.2) | 149.2 (109.5-200.2) | 192.7 (146.8-260.2) | <0.001 |  | 161.5 (122.2-235.6) | 167.3 (127.6-217.1) | 0.768 |
| **TWEAK** | 215.5 (166.2-309.6) | 196.4 (159.9-251.6) | 195.9 (155.2-245.2) | 0.090 |  | 198.2 (159.9-248.6) | 198.6 (158.0-254.1) | 0.259 |
| **PTH** | 59.1 (44.5-76.9) | 63.7 (49.4-75.8) | 74.2 (51.6-94.2) | <0.001 |  | 74.0 (54.1-89.5) | 61.2 (47.3-78.9) | 0.027 |
| **Phosphate** | 3.3 (3.0-3.6) | 3.3 (2.8-3.6) | 3.2 (2.8-3.7) | 0.528 |  | 3.4 (3.1-3.8) | 3.2 (2.8-3.5) | 0.003 |
| **FGF23** | 64.5 (50.2-81.5) | 61.8 (48.4-84.3) | 78.8 (60.2-107.9) | 0.271 |  | 77.2 (59.4-107.0) | 65.0 (53.3-85.9) | 0.221 |
| **Calcidiol** | 18.6 (11.7-249) | 18.5 (12.5-24.5) | 18.0 (12.5-21.1) | 0.980 |  | 16.6 (11.9-24.0) | 18.8 (12.6-25.2) | 0.194 |

**S1 Table:** Levels of the different biomarkers distributed by age and sex:

**Abbreviations as for Table 1.**
