# Supplementary material for: Plasma Levels of Monocyte Chemoattractant Protein-1, n-Terminal Fragment of Brain Natriuretic Peptide and Calcidiol Are Independently Associated with the Complexity of Coronary Artery Disease
Source: PLoS One. 2016 May 12;11(5):e0152816. doi: 10.1371/journal.pone.0152816 (PMC4865225; doi:10.1371/journal.pone.0152816)
Supplement: S2 Table — (DOCX) [file pone.0152816.s002.docx]

**S2 Table:** Univariate linear regression analysis for prediction of Syntax Score:

| **Variable** | **p Value** |  | **Variable** | **p Value** |
| --- | --- | --- | --- | --- |
| Age | **0.000** |  | AP | 0.664 |
| Gender | **0.026** |  | LDL-c | 0.948 |
| Hypertension | **0.000** |  | HDL-c | **0.004** |
| Diabetes | **0.025** |  | STEMI | 0.969 |
| Hyperlipidemia | 0.104 |  | Tryglicerides | 0.064 |
| Smoker | 0.354 |  | Calcidiol | 0.254 |
| ASA | 0.085 |  | Phosphate | 0.854 |
| Clopidogrel | 0.962 |  | FGF-23 | 0.987 |
| Acenocumarol | 0.876 |  | PTH | 0.090 |
| Statins | 0.069 |  | hs-CRP | 0.104 |
| ACEI | 0.700 |  | Galectin-3 | 0.071 |
| ARB | 0.715 |  | MCP-1 | **0.001** |
| β-Blockers | 0.125 |  | NGAL | **0.002** |
| BMI | 0.500 |  | sTWEAK | 0.052 |
| eGFR: | **0.000** |  | NT-proBNP | **0.000** |

**Abbreviations as for Table 1.**
