# Supplementary material for: Plasma Levels of Monocyte Chemoattractant Protein-1, n-Terminal Fragment of Brain Natriuretic Peptide and Calcidiol Are Independently Associated with the Complexity of Coronary Artery Disease
Source: PLoS One. 2016 May 12;11(5):e0152816. doi: 10.1371/journal.pone.0152816 (PMC4865225; doi:10.1371/journal.pone.0152816)
Supplement: S3 Table — (DOCX) [file pone.0152816.s003.docx]

**S 3 Table:** Univariate logistic regression analysis for prediction of Coronary Artery Calcification:

| **Variable** | **p Value** |  | **Variable** | **p Value** |
| --- | --- | --- | --- | --- |
| Age | **<0.001** |  | AP | 0.868 |
| Gender | 0.193 |  | LDL-c | 0.532 |
| Hypertension | 0.063 |  | HDL-c | 0.548 |
| Diabetes | **0.045** |  | STEMI | **0.005** |
| Hyperlipidemia | 0.641 |  | Tryglicerides | 0.939 |
| Smoker | 0.172 |  | Calcidiol | **0.006** |
| ASA | 0.200 |  | Phosphate | 0.351 |
| Clopidogrel | 0.761 |  | FGF-23 | 0.432 |
| Acenocumarol | 0.988 |  | PTH | **0.042** |
| Statins | 0.303 |  | hs-CRP | 0.947 |
| ACEI | 0.079 |  | Galectin-3 | 0.320 |
| ARB | 0.929 |  | MCP-1 | **0.029** |
| β-Blockers | 0.751 |  | NGAL | **0.005** |
| BMI | 0.579 |  | sTWEAK | 0.263 |
| eGFR: | **0.007** |  | NT-proBNP | **0.032** |

**Abbreviations as for Table 1.**
