# Supplementary material for: Plasma Levels of Monocyte Chemoattractant Protein-1, n-Terminal Fragment of Brain Natriuretic Peptide and Calcidiol Are Independently Associated with the Complexity of Coronary Artery Disease
Source: PLoS One. 2016 May 12;11(5):e0152816. doi: 10.1371/journal.pone.0152816 (PMC4865225; doi:10.1371/journal.pone.0152816)
Supplement: S4 Table — (DOC) [file pone.0152816.s004.doc]

**S4 Table: Univariate Cox regression analysis for prediction of AAE**

| **Variable** | **p Value** |  | **Variable** | **p Value** |
| --- | --- | --- | --- | --- |
| Age | **0.041** |  | Atrial Fibrillation | 0.818 |
| Gender | 0.097 |  | Aspirin | 0.915 |
| Hypertension | **0.017** |  | Clopidogrel | 0.520 |
| Diabetes | 0.362 |  | Statins | 0.599 |
| Dyslipidemia | 0.967 |  | ACEI | 0.146 |
| Smoker | **0.035** |  | ARB | **0.037** |
| Body mass index (kg/m^2^) | 0.057 |  | β-Blockers | **0.037** |
| LVEF<40% | 0.158 |  | Calcidiol | 0.260 |
| Type of ACS | 0.671 |  | Phosphate | 0.143 |
| eGFR | **0.035** |  | FGF-23 | 0.428 |
| Syntax Score | **0.016** |  | PTH | 0.211 |
| BC | **0.008** |  | MCP-1 | **0.019** |
| Combined SS / Calcium score | **0.006** |  | NGAL | 0.300 |
| Platelet count | 0.570 |  | sTWEAK | 0.361 |
| Leucocytes count | 0.277 |  | Hs-CRP | 0.424 |
| Cerebrovascular events | 0.693 |  | Galectin-3 | **0.017** |
| Peripheral artery disease | 0.429 |  | NT-proBNP | 0.341 |

**Abbreviations as for Table 1.** P value was calculated by likelihood ratio method.
